# The mysterious ecosystem at the ocean’s surface

**DOI:** 10.1371/journal.pbio.3001046

**Published:** 2021-04-28

**Authors:** Rebecca R. Helm

**Affiliations:** 1 Department of Biology, University of North Carolina Asheville, Asheville, North Carolina, United States of America; 2 Invertebrate Zoology, Smithsonian National Museum of Natural History, Washington, DC, United States of America

## Abstract

Life on the ocean’s surface connects worlds. From shallow waters to the deep sea, the open ocean to rivers and lakes, numerous terrestrial and marine species depend on the surface ecosystem and the organisms found therein. Organisms that live freely at the surface, termed “neuston,” include keystone organisms like the golden seaweed *Sargassum* that makes up the Sargasso Sea, floating barnacles, snails, nudibranchs, and cnidarians. Many ecologically and economically important fish species live as or rely upon neuston. Species at the surface are not distributed uniformly; the ocean’s surface harbors unique neustonic communities and ecoregions found at only certain latitudes and only in specific ocean basins. But the surface is also on the front line of climate change and pollution. Despite the diversity and importance of the ocean’s surface in connecting disparate habitats, and the risks it faces, we know very little about neustonic life. This Essay will introduce you to the neuston, their connections to diverse habitats, the threats they face, and new opportunities for research and discovery at the air-sea interface.

## Abstract 2

The mysterious ’neuston’ ecosystem at the ocean’s surface includes keystone organisms like the golden seaweed Sargassum that makes up the Sargasso Sea, floating barnacles, snails, nudibranchs, and cnidarians; this Essay explores threats to its wellbeing and the importance of further research.

## Introduction

The ocean’s surface acts like a skin between the atmosphere above and the water below, and harbors an ecosystem unique to this environment. This sun-drenched habitat can be defined as roughly 1 meter in depth, as nearly half of UV-B is attenuated within this first meter [1]. Organisms here must contend with wave action and unique chemical [2–5] and physical properties [4]. The surface is utilized by a wide range of species, from various fish and cetaceans, to species that ride on ocean debris (termed rafters) [6–8]. Most prominently, the surface is home to a unique community of free-living organisms, termed “neuston” (from the Greek word, υεω, which means both to swim and to float. Floating organisms are also sometimes referred to as pleuston, though neuston is more commonly used).

**Fig 1:**
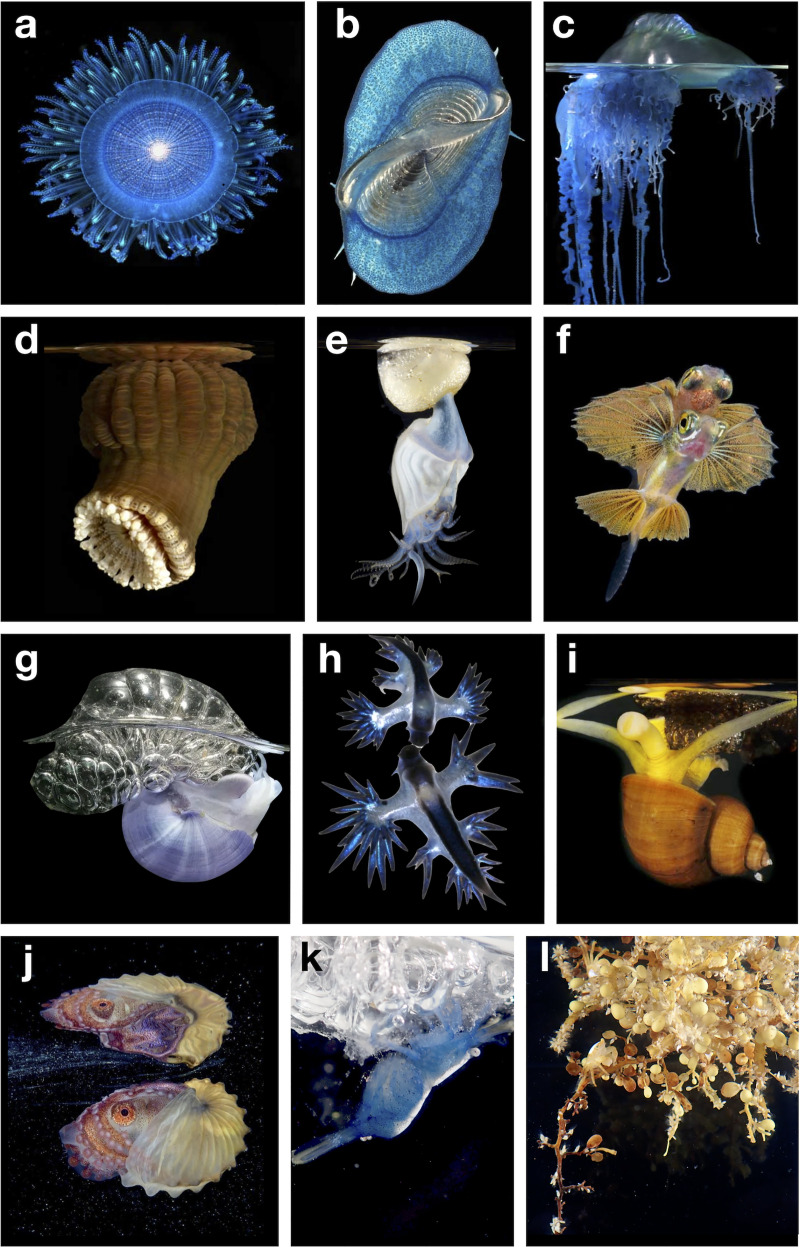
Diverse members of the ocean surface ecosystem. (a) Blue button *Porpita* sp. viewed from above, (b) by-the-wind sailor *Velella* sp. viewed from above, (c) Portuguese man-o-war *Physalia* sp. viewed from the side, with the float above the surface, (d) the floating anemone *Actinecta* sp. viewed from the side, with the aboral float at the surface, (e) buoy barnacle *Dosima fascicularis* viewed from the side, with aboral white float at the water’s surface, (f) a young flying fish (family Exocoetidae) viewed from below, reflected in the surface above, (g) violet snail *Janthina* sp. viewed from the side, with a large bubble raft made from snail mucus emerging from the water, (h) blue sea dragons *Glaucus* sp. viewed from above with dark blue ventral surfaces, (i) the snail *Recluzia* sp. viewed from the side oral end, (j) paper nautilus *Aurgonaut* sp. viewed from the side and reflecting off the water’s surface, (k) a shrimp in the family Hippolytidae, clinging to a discarded *Janthina* bubble raft, (l) seaweed *Sargassum* sp. with a small sargassum crab *Portunus sayi*. Images a–e and g–i by Denis Riek, f and j by Songda Cai, k and l by Rebecca R. Helm.

**Fig 2:**
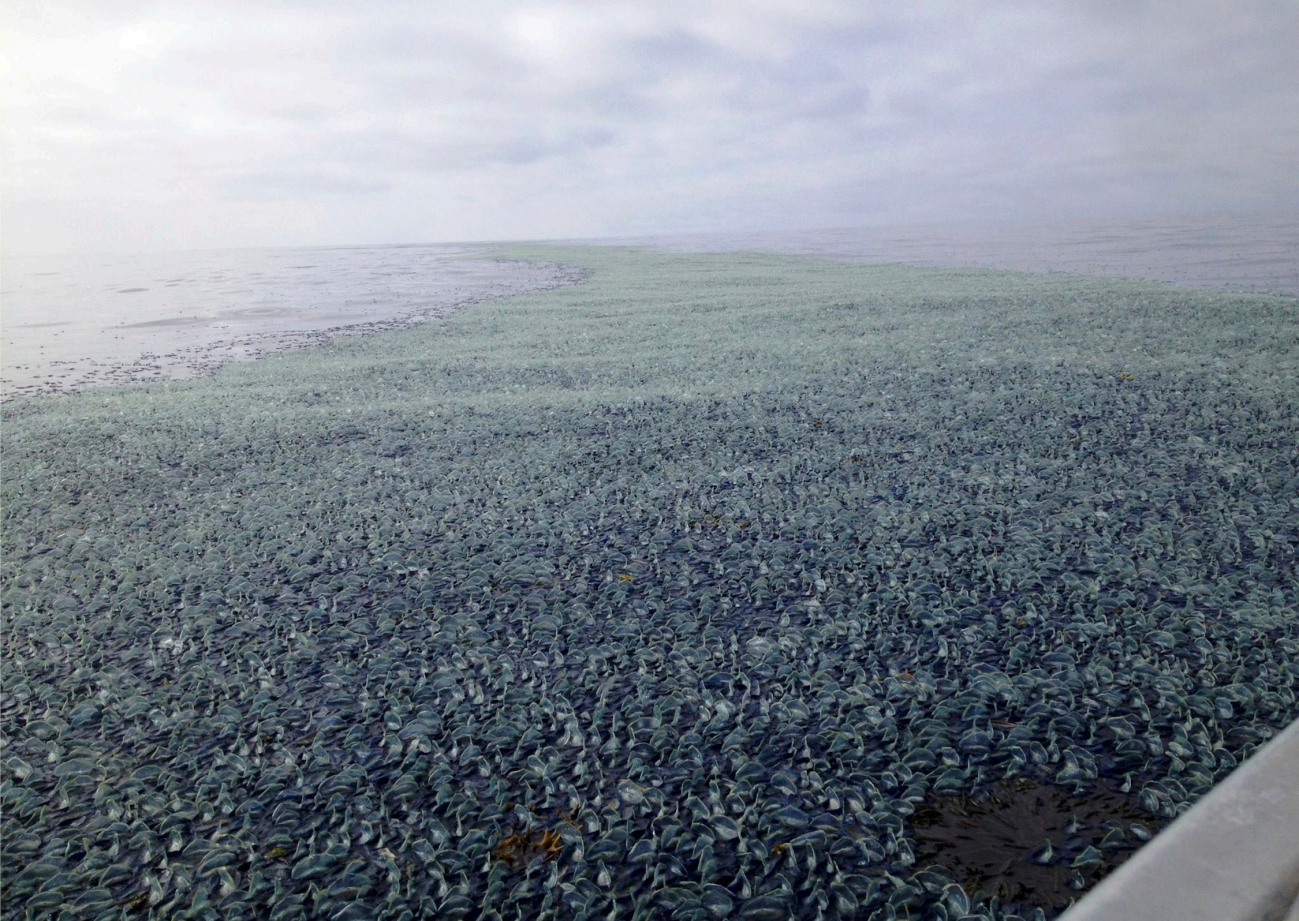
Neuston on the ocean’s surface. By-the-wind sailors *Velella* sp. covering the ocean’s surface off the coast of La Push, Washington State, United States of America. Image by Scott Horton.

Neuston (Fig 1) are key ecological links connecting ecosystems as far ranging as coral reefs, islands, the deep sea, and even freshwater habitats. In the North Pacific, 80% of the loggerhead turtle diet consists of neuston prey [9], and nearly 30% of the Laysan albatross’s diet is neuston [10]. Diverse pelagic and reef fish species live at the surface when young [11] (Table 1), including commercially important fish species like the Atlantic cod (*Gadus* spp.), salmon (*Oncorhynchus*), and billfish (Istiophoriformes). Neuston can be concentrated as living islands that completely obscure the sea surface (Fig 2), or scattered into sparse meadows over thousands of miles. Yet the role of the neuston, and in many cases their mere existence, is often overlooked.

**Table 1:** Select fish species that depend on the ocean’s surface for food or egg/larval habitat.

Adult habitat	Common name	Taxonomy	References
**Pelagic open ocean**	Marlins	Istiophoridae	[11,38–42]
	Swordfish	Xiphiidae	[11,38,42,43]
	Anchovy	Engraulidae	[37,38,44,45]
	Dolphinfish (including Mahi-mahi)	Coryphaenidae	[11,38,40,45–47]
	Diverse flying fish	Exocoetidae	[11,38,48,49]
	Amberjack	*Seriola dumerili*	[11,40]
	Atlantic mackerel	*Scomber scombrus*	[22,50,51]
**Nearshore**	Mullet	Mugilidae	[40,44,52,53]
	Bluefish	Pomatomidae	[44,45,49]
	Lefteye flounders	Bothidae	[44,45,49]
	Atlantic cod	*Gadus morhua*	[22,45]
**Deep sea**	Viperfish	*Chauliodus*	[37,46]
	Lanternfish	Myctophidae	[38,54,55]
	Oarfish, ribbonfishes, etc.	Lampriformes	[37,46]
**Reef**	Seahorses, seadragons and pipefishes	Syngnathidae	[38,44,45]
	Damselfishes and clownfishes	Pomacentridae	[11,53,54,56]
	Blennies	Blenniidae	[53,54,56]
**Fresh and salt water (anadromous)**	Chinook (king) salmon	*Oncorhynchus tshawytscha*	[57–60]
	Coho salmon	*Oncorhynchus kisutch*	[57–60]
	European eel	*Anguilla anguilla*	[61,62]
	American eel	*Anguilla rostrata*	[45,61,63,64]

One of the most well-known surface ecoregions is the Sargasso Sea, an ecologically distinct region packed with thick, neustonic brown seaweed in the North Atlantic. Multiple ecologically and commercially important species depend on the Sargasso Sea, but neustonic life exists in every ocean basin and may serve a similar, if unrecognized, role in regions across the planet. For example, over 50 years ago, USSR scientist A. I. Savilov characterized 7 neustonic ecoregions in the Pacific Ocean [12]. Each ecoregion possesses a unique combination of biotic and abiotic conditions and hosts a unique community of neustonic organisms. Yet these ecoregions have been largely forgotten.

But there is another reason to study neuston: The ocean’s surface is on the front line of human impacts, from climate change to pollution, oil spills to plastic. The ocean’s surface is hit hard by anthropogenic change, and the surface ecosystem is likely already dramatically different from even a few hundred years ago. For example, prior to widespread damming, logging, and industrialization, more wood may have entered the open ocean (as an example, see [13]), while plastic had not yet been invented. And because floating life provides food and shelter for diverse species, changes in the surface habitat will cause changes in other ecosystems and have implications that we may not fully understand or be able to predict.

Studying life at the ocean’s surface is a global challenge. This is a job that no one person or research group can accomplish alone. It will take both professional scientists and passionate naturalists to unlock the mysteries of this unique and stunning ecosystem and the role it plays in the health and diversity of Earth’s oceans.

To promote research on the ocean’s surface ecosystems, I provide an overview of neuston ecology and the potential impacts the neuston may face. In addition, I propose key areas of research and observation that can help unravel the mysteries of this unique ecosystem. (See S1 Text for a guide to free-living ocean surface life.)

## How is the ocean’s surface connected to ecosystems both above and below the waves?

*“Just before it was dark*, *as they passed a great island of Sargasso weed that heaved and swung in the light sea as though the ocean were making love with something under a yellow blanket*, *his small line was taken by a dolphin.”*—*Ernest Hemingway, The Old Man and the Sea*.

### Ecoregions

The ocean’s surface possesses diverse floating ecosystems within different regions. The only well-known neustonic ecoregion, the Sargasso Sea, covers an area in the western North Atlantic where the neustonic *Sargassum* concentrates. Multiple endemic species live in the Sargasso Sea, many of them adapted to shelter among the neustonic seaweed [14,15]. The Sargasso Sea contributes to a variety of ecosystem goods and services, and its valuation ranges from over US$200 million for fisheries services to US$2.7 billion for all services [16]. The distribution of surface life in the Sargasso Sea changes by season [17,18] and may be subject to annual and decadal trends [19,20]. These trends can impact both the ecology and economy of the region, as well as stakeholders further afield that rely on species that shelter in the *Sargassum*.

Beyond the Sargasso Sea, the most detailed survey of Pacific neuston occurred in the 1950s. Scientists in the USSR crisscrossed the Pacific, collecting nearly 500 samples from 50°N to over 40°S [12]. In this massive survey, 7 distinct ecoregions were discovered, with different species of neuston showing different ranges that likely reflect their ability to move with wind, thermal optima, seasonality, and life cycles [12].

A linear survey from Fiji to the Bay of Biscay also found considerable geographic variation. The tropical seas of the Indian Ocean were dominated by neustonic species including *Halobates*, *Physalia*, *Velella*, and *Porpita* [21]. In contrast, the eastern North Atlantic was dominated by small quick-moving crustaceans that made up over 90% of neustonic organisms [21,22]. This suggests these regions have distinct neustonic communities.

Ecological variation across regions also includes how the surface changes through time. On short time scales, the surface habitat is part of the diel vertical migration of marine life from the deep sea: the largest migration on Earth, which happens twice each day [23]. Because of this migration, significant differences in surface life occur between day and night at basin-wide scales. Ostracods, mysids, isopods, heteropods, various crustacean and bryozoan larvae, are all more abundant at the surface at night [21,22]. In contrast, some surface-associated species, such as *Sapphirina* copepods, which use complex visual cues for mating, migrate to the surface only during the day [24]. These migratory species add to the diversity at the ocean’s surface. On larger time scales, neustonic *Sargassum* abundance changes seasonally [19,25,26], and some neuston, such as *Velella*, strand more often in certain seasons than others [27], possibly due to seasonal variation in distribution.

Differences in neuston across space and time may be due to real population and species boundaries. For example, while some species, such as the nudibranch *Glaucus atlanticus*, are globally distributed, closely relatives *Glaucus bennettae* and *Glaucus mcfarlanei* have thus far been identified only in the North Pacific subtropical gyre system [28], and represent cryptic species. The sea skater *Halboates* shows remarkable population- and species-level isolation both across oceans and ocean basins [29,30], while neustonic *Sargassum* represent a genetic and morphotype species complex with diverse and distinct distribution patterns [31]. It is clear that neuston are not uniformly distributed, and there is evidence for both species and population isolation as well as sympatric speciation. However, for the majority of neustonic species, no genetic or population data exist. Are individuals of the “same species” half a planet away part of an interconnected global population, or isolated and distinct enough to be considered different species with unique adaptations to the conditions in their region of the world?

Poorly studied neuston ecoregions should be considered in the context of the Sargasso Sea: We know this comparatively well-studied region is critical for both the ecology and economy of the North Atlantic, its services valued in the billions. What ecological and economic services are neuston ecosystems providing in other ocean regions?

### Food webs

**Fig 3:**
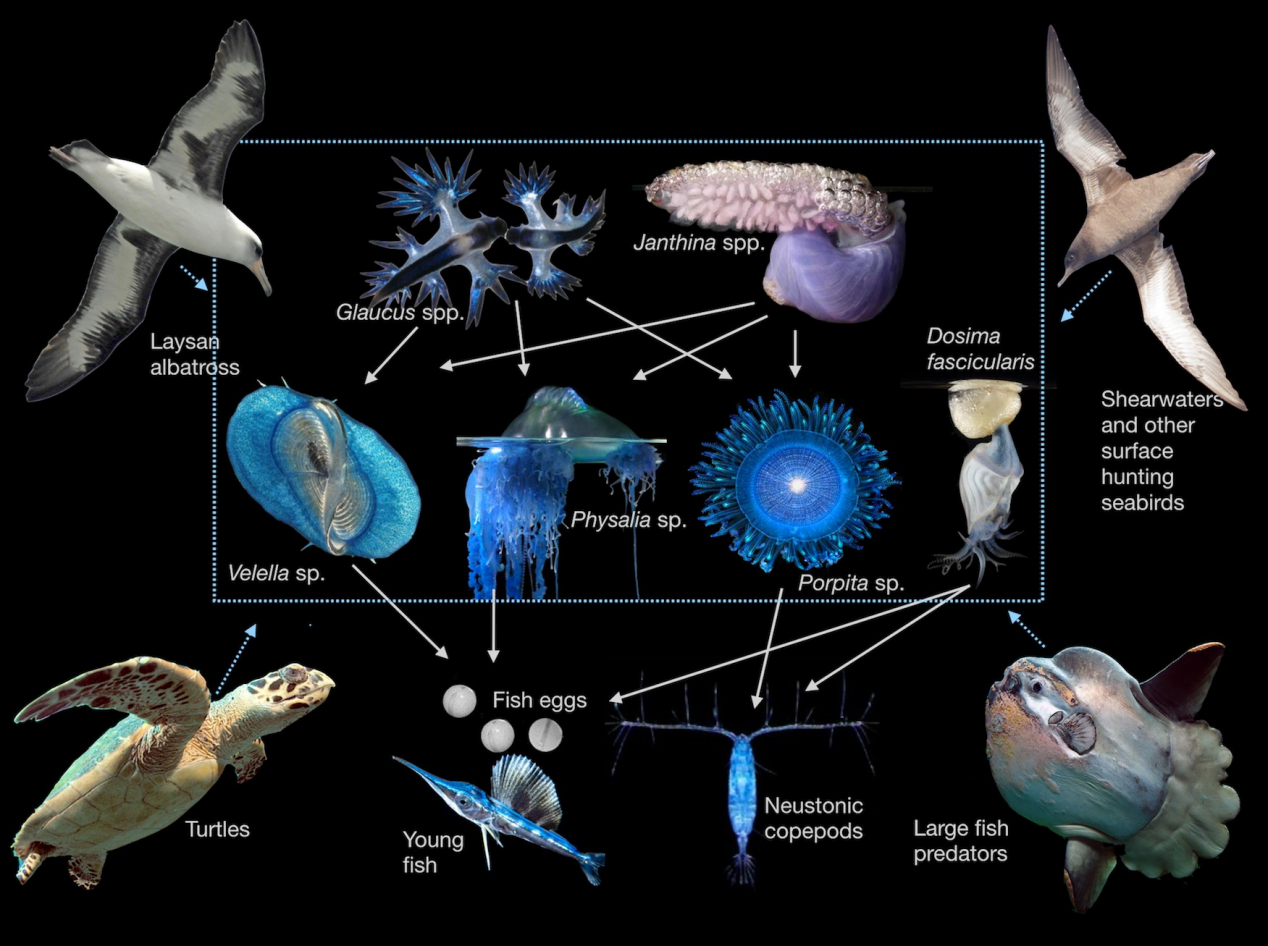
The neuston food web. A simplified surface food web based on [65], with floating species in the grey box, which may be preyed upon similarly by large predators (though see [65,66] for distinguishing features of each species). Images of *Mola mola*, Laysan albatross, hawksbill sea turtle, and sooty shearwater from Wikimedia Commons. Images of *Glaucus marginatus*, *Janthina umbilicata*, *Dosima fascicularis*, *Physalia* sp., *Velella* sp., and *Porpita* sp. by Denis Riek. Image of copepod from [67], image of fish eggs from [68], image of young sailfish by Linda Ianniello.

Organisms that live at the surface are a nexus for food webs both above and below (Fig 3). From the air, seabirds prey on neuston, including fulmars, storm petrels, and sooty shearwaters (see review in [32]). For the Pacific ocean Laysan albatross, nearly 30% of their diet is neuston, including *Velella*, *Janthina*, *Halobates*, and the eggs and larvae of flying fish [10]. Even ducks [33] and sea-going bats [34] prey on floating neuston when they drift close to shore. Below the surface, diverse sea turtles eat neuston (see review in [32]), including olive ridley (*Lepidochelys olivacea*), which prey upon *Janthina* [9,35], green turtles (*Chelonia mydas*), which prey upon *Porpita* [36], and loggerhead turtles (*Caretta caretta*), which prey upon *Velella* and *Janthina* [9]. Neuston are among the most important prey for central North Pacific loggerhead sea turtles [9]. Fish like coho salmon *Oncorhynchus kisutch* and spiny dogfish *Squalus acanthias* prey on *Velella* (see review in [32]), and animals of the deep-scattering layer also prey upon neuston [37]. Diverse larval fish from a wide variety of ecologically and economically important species live as or prey on neuston [11] (Table 1).

Neuston themselves reach into the waters below, capturing non-neustonic prey and further linking deeper waters to this thin surface layer. *Velella* feed on a variety of foods, including fish eggs and larvae [69], while *Porpita* and *Dosima fascicularis* consume fast-moving carnivorous calanoid copepods [66,70]. Unlike *Velella* and *Porpita*, which each have tentacles extending only a few centimeters, *Physalia* can extend tentacles many meters below the surface, and prey primarily on fish [71].

Many species of the neuston also prey on one another, creating an interconnected food web stretching into the broader world around it. *Janthina* and *Glaucus* prey on *Physalia*, *Velella*, and *Porpita*. *Janthina* have also been observed trying to eat each other, suggesting they have the capacity to be cannibalistic [65]. The only true open ocean insect, the neustonic *Halobates*, preys upon other neuston by sucking nutrients from organisms with piercing mouthparts [72].

### Life history

Life histories connect disparate ecosystems; species that live at the surface during one life history stage may occupy the deep sea, benthos, reefs, or freshwater ecosystems during another. A diversity of fish species utilize the ocean’s surface [73], either as adults or as nursery habitat for eggs and young (examples in Table 1). In contrast, species floating on the ocean’s surface during one life cycle stage often (though not always) have pelagic larval stages. *Velella* and *Porpita* release jellyfish (medusae) [74], and while we know very little about *Porpita* medusae, *Velella* medusae could possibly sink into deeper water [74], or remain near the surface, where they derive nutrients from zooxanthellae [75]. *Janthina* have pelagic veliger larvae [76], and *Physalia* may release reproductive clusters that drift in the water column. *Halobates* lay eggs on a variety of objects, including floating objects [72] and pelagic snail shells [77].

All species with pelagic stages must eventually find their way back to the surface. For *Velella* and *Porpita*, larvae generated by sexual reproduction of medusae develop small floats, which carry them to the surface [78,79]. For the larvae of *Janthina*, the transition to surface life includes the degradation of their eyes and vestibule system, and at the same time, the production of an external structure, which has been reported as either a small parachute made of mucus, or a cluster of bubbles, which they ride to the surface [80,81]. Young *Halobates* may hatch either above or below the surface, and for those below, the surface tension proves a formidable barrier. It may take *Halobates* nymphs several hours to break through the surface film [77]. Despite the challenges of reaching the surface, there may be benefits to a temporary pelagic life.

**Fig 4:**
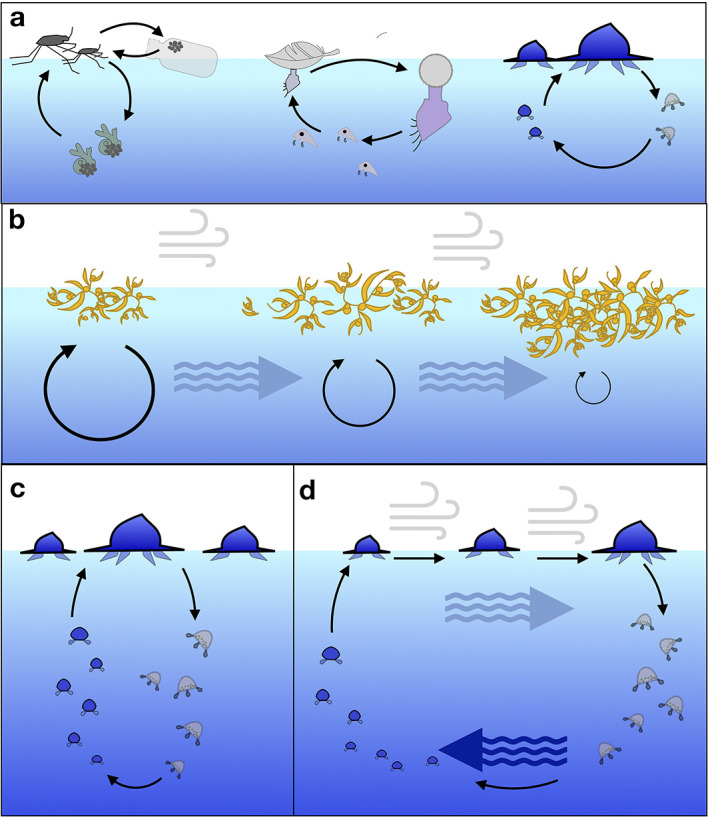
Possible life history mechanisms for localization and dispersal of neustonic organisms. (a) Some neustonic species lay eggs on floating objects and sometimes pelagic organisms (e.g., *Halobates* spp.), while others require surface floating objects for early life cycle stages (e.g., *Dosima fascicularis* [83]), still others may remain at or near the surface throughout a life cycle due to a dependence on endosymbiotic photosynthetic zooxanthellae (a hypothesis proposed by Larson for *Velella* [75]). (b) Neustonic organisms like *Sargassum* may proliferate in one region (large circle) and be transported by wind and/or currents to high-density regions of low proliferation (small circles) [25]. (c) Neuston may also occupy deep water for one part of their life history (a hypothesis proposed for *Velella* by Woltereck [84]), and (d) these deep-water habitats may allow them to take advantage of counter currents for transport in the direction opposite surface currents (a hypothesis proposed for *Velella* by Savilov [12]).

Connectivity of ocean surface ecosystems may be facilitated by the life history of species living there (Fig 4). One hypothesis is that species have pelagic stages to “escape” surface sink regions and repopulate surface source regions, where one life cycle stage drifts on surface currents in one direction, and a pelagic stage either remains geographically localized [82] or drifts in the opposite direction [12]. However, some surface species, such as the endemic species of the Sargasso Sea, may remain geographically isolated throughout their life history. While these hypotheses are intriguing, we do not know if or how life history shapes population/species distribution for most neustonic species. Understanding how life history varies by species is a critical component of assessing both connectivity and conservation of neustonic ecosystems.

## What threats does the ocean’s surface face?

The ocean surface is a concentrating front for floating pollutants from plastic to petroleum. Metals and toxicants concentrate on the ocean’s surface, particularly hydrophobic molecules such as aromatic hydrocarbons, pesticides, and polychlorinated biphenyls (PCBs), which can all have sublethal and lethal impacts on larval fish [85]. In addition, chlorinated and petroleum hydrocarbons, organotin compounds, polycyclic aromatic hydrocarbons (PAH), and heavy metals at the surface can reach concentrations up to 500 times higher than those in the water column [86]. Many of these compounds are concentrated in the sea surface microlayer (0 to 1,000 μm depth). In general, pollutants are at lower concentrations in the open ocean than in areas closer to shore [86], and while this may bode well for open-ocean neustonic species, it presents challenges to coastal or benthic species with neustonic eggs or larvae (Table 1).

One large threat to both coastal and open-ocean surface organisms comes from oil. An estimated 741 kilotonnes of oil is released into the ocean each year from both natural and human sources [87], with unknown effects on surface ecosystems. Because hydrophobic molecules concentrate at the ocean’s surface [2,88], neustonic species will face orders of magnitude higher oil exposure than animals even a meter below the surface. Additionally, neuston species may also be vulnerable to dispersants used in breaking down oil spills, as is the case with jellyfish [89], which die at significantly higher rates in the presence of dispersants, and *Sargassum*, which sinks in the presence of dispersants [90]. However, studies on diverse neustonic species in the presence of oil or dispersants have not been conducted.

Floating plastic is another widespread petroleum product on the ocean’s surface [91,92]. There are an estimated 14.9 to 51.2 trillion pieces of plastic on the ocean’s surface [93], representing upwards of 250,000 tons, largely concentrated in oceanic subtropical gyres (known colloquially as “Garbage Patches,” which includes the Sargasso Sea) [94]. These plastics are consumed by surface-hunting species like the Laysan albatrosses of Midway Atoll, which feed nearly 5 tons of ocean plastic to their chicks each year [95,96]. Such high plastic consumption makes sense only in light of these birds’ predation on neuston [10]. Larval neustonic fish and rafting barnacles have been found with plastic in their gut [11,97], though the impact of this plastic on these organisms, or the animals that feed on them, is not known. Some neustonic species, such as *Halobates*, may benefit from plastic, which provides a hard surface for laying eggs [98]. Larval fish may also shelter around plastic debris [73].

With this complexity in mind, we must proceed cautiously when attempting to restore or conserve the ocean’s surface. For example, multiple organizations pledge to remove plastic from the ocean using unmanned collection devices inspired by pool skimmers or technology used to catch algae and jellyfish [99]. It should be no surprise then that one organization trapped hundreds of neustonic animals in their prototype, visible in their press release photo [100].

Of all the human impacts on the ocean’s surface, climate change will have the farthest reach, and it is unclear what impact it will have on neuston. The ocean’s surface is directly exposed to the atmosphere, and changes in temperature will be felt first at the surface. This region is also uniquely exposed to atmospheric carbon dioxide and the ravages of storms, which are predicted to increase in intensity and frequency under climate change [101].

Overall, threats to the neuston are poorly understood, and for every likely threat listed here, there are no doubt many that are largely unknown (e.g., ballast water, localized pollution, deep-sea mining impacts on pelagic life-cycle stages, geoengineering, etc.).

Without a better understanding of the neuston, the best ways to preserve and protect the ocean’s surface are far from clear. For example, changes in surface-associated fish populations (e.g., billfish, mahi-mahi, salmon, etc.) or increases in sea turtles or sea bird mortality may all stem from acute changes in the ocean’s neustonic ecosystem. With the right initiative, these changes can be monitored, mitigated, or even reversed. Understanding the dynamics of the ocean’s surface is truly a challenge of global proportions. The surface knows no national boundaries; indeed, areas where there are likely high concentrations of open-ocean neuston (like subtropical gyres) are found beyond national jurisdiction. No one person or group can fully understand or regulate the health of this vital ecosystem. Fortunately, we can think bigger.

## What actions can we take to better understand the ocean’s surface?

Below I have identified some key areas of research and community action that will contribute to our understanding and conservation of the ocean’s surface.

1. **Community monitoring:** A global community monitoring network for reporting the presence and absence of neuston and associated strandings will provide much-needed baseline data on when and where species occur, and in what abundance. This work will require a global effort linking scientists to community members and organizations (community or citizen science), to report the presence and absence of organisms to organizations like iNatralist.org or JellyWatch.org. The importance of this work cannot be understated: Without accurate predictive tools for when and where neuston occur, we cannot study them, and without basic data on their distribution, we cannot generate predictive tools. With these data, we can begin to address critical questions in surface biology.
2. **Identifying open ocean regions of high importance:** The open ocean’s surface is not uniform, and we must identify ocean regions that provide high ecosystem services. The Sargasso Sea is a key example of a critical surface ecosystem, but it may be far from the only one. The Sargasso Sea is located on the western edge of the North Atlantic Gyre, and there are 5 subtropical gyres globally. All subtropical gyres concentrate floating plastic (including the Sargasso Sea [94]). If all gyres concentrate floating plastic, it stands to reason they may also concentrate neustonic life. Other subtropical gyre regions and regions of increased plastic concentration (e.g., oceanic convergence zones) should be investigated for high densities of surface life. On a relatively small scale, surface slicks may also be critical in concentrating neuston [73]. These regions may contain both high biomass and biodiversity of neuston and may be important for species that depend on the ocean’s surface habitat. Physical surveys (e.g., [102]) and modeling studies are necessary to understand how neuston distribution varies over both large and small spatial scales.
3. **Population connectivity, life history, and resource webs:** Understanding population connectivity and species distribution is an important component of characterizing sources and sinks, the role species distribution plays in replenishing new regions, and the impact industries may have on open ocean neuston ecosystems. We must study the role of population connectivity and transport using distribution data, modeling, and genetic data. Due to the complex transport at the ocean’s surface, some ocean regions may act as sources for neuston, while others as sinks. For example, the Sargasso Sea may be a *Sargassum* dead end [25]. Conservation of the Sargasso Sea is an important first step in protecting the services it provides, but must be done with a clear understanding of the ways other regions contribute. To this point, areas of high neuston concentration and regional connectivity are likely impacted by neuston life history. A basic understanding of neuston life cycles is necessary to understand species distribution and their connections to other ecosystems. Likewise, understanding the food web and interdependence of neustonic ecosystems will make it possible to identify “keystone” species that are critical for ecosystem function. While neuston form the core of the surface ecosystem, they are far from alone. Whales visit the surface to breath, sea birds to feed, rafting organisms ride on floating debris. Understanding the ecology of the surface means studying both the abiotic conditions of the atmosphere and ocean, and the interdependence of all organisms that utilize this remarkable habitat.
4. **Understanding the economy of the ocean’s surface:** Commercially important fish like anchovy, marlin, salmon, Atlantic cod, and mahi-mahi all utilize the surface (Table 1). Diverse salmon and billfish species rely on the surface either for habitat or food, and the commercial value of these two fisheries alone represents over US$9,000 million, and support nearly 40,000 jobs [103,104]. And our economic dependence on the ocean’s surface has a deep history: The now critically endangered European eel, which spawns in the Sargasso Sea before swimming upstream in Europe to live in freshwater, was once used as currency for paying rent in medieval England (John Wyatt Greenlee, personal communication, 2020). These are just a few examples of the direct link between the open-ocean surface and diverse ecologies and economies. And we do not understand how changes in neustonic communities could, or are, affecting fisheries.
5. **Impacts at the ocean’s surface:** The consequences of plastics, oil spills, pollution, fishing, and climate change are likely substantial for the ocean’s surface but have only been evaluated for a small number of neustonic species (e.g., [11,90,98]). Because the air-sea interface is subject to unique chemical and physical properties, impact studies from other marine ecosystems may not be translatable to this habit. For example, plastic may “increase” certain neustonic species by providing habitat [98], and oil dispersants may have a more severe impact on surface life than the oil itself [11,90]. This is not to say there are no risks from plastic or oil, but instead that the risks are complex and distinct for the ocean’s surface. For this reason, we must study impacts on the neuston rather than assume their outcome based on other marine systems.
6. **Legal protection of the ocean’s surface:** Understanding where neustonic organisms concentrate, their food web dynamics, population connectivity, species boundaries, and commercial value is critical to conserving this habitat, but is not sufficient without legal protection. Protecting surface ecosystems will preserve their functions and buffer them against exploitation. Because many of these regions are beyond areas of national jurisdiction, this will require a coordinated international effort. Legal requirements for assessing and monitoring environmental impacts are severely lacking in international waters, but it is essential that the ocean surface ecosystem be considered, especially where a significant surface impact is possible, including (but not limited to): oil, natural gas, sediment pollution, plastic pollution, and unmonitored ocean-surface objects or nets (including plastic interceptors, large scale boats collecting plastic, fishing, etc.). A clear plan should be reviewed and agreed upon by an international authority to assess these impacts based on the input of scientists and conservation stakeholders. And for both protected areas and areas of potential high human impact, there should be clear protocol for monitoring and managing activities, enforcing regulations, and holding organizations and governments accountable. Given how little we know about the surface ecosystem, a conservative and precautionary approach should be taken.

The ocean’s surface is truly a global resource, one that connects diverse ecosystems and provides key services to our world that we are only beginning to understand. To protect the valuable role the ocean’s surface plays on our planet, we must research and conserve this remarkable habitat between the sea and sky.

## Supporting information

S1 Text Supplemental guide to common neustonic organisms found at the ocean’s surface. (PDF)
