# Supplementary material for: The mysterious ecosystem at the ocean’s surface
Source: PLoS Biol. 2021 Apr 28;19(4):e3001046. doi: 10.1371/journal.pbio.3001046 (PMC8081451; doi:10.1371/journal.pbio.3001046)
Supplement: S1 Text — (PDF) [file pbio.3001046.s001.pdf]

The Mysterious Ecosystem at the Ocean's Surface: Guide to Ocean Surface Life

Rebecca R. Helm[1,2,*]

1 Department of Biology, University of North Carolina Asheville, Asheville, NC, United States of America
2 Invertebrate Zoology, Smithsonian National Museum of Natural History, Washington, DC, United States of America
* rrhelm@gmail.com

1 Guide

Invoking images of the open ocean's surface, the imagination often conjures up an endless empty space. A flat line parting the blue below from the blue above. But in reality a diverse array of species occupy this unique boundary layer. A tangle of terms exist for different organisms occupying different niches of the ocean's surface. For the purposes of this Essay, I have used the most inclusive term, neuston, to refer to all of them. In this guide I include more specific terms for each functional group, though it should be noted that organisms may move between groups depending on their life cycle stage.

1.1 Organisms which float at the surface (aka pleuston)

Many organisms have morphological features that enable them to remain at the ocean's surface, with the most noticeable adaptations being floats. Neustonic animals that live floating at the ocean's surface are sometimes referred to as pleuston. They live hanging from the surface of the ocean as if suspended from the roof of a massive cave, and are incapable of controlling their direction of movement. They are considered permanent residents of the surface layer. Many genera are globally distributed.

1.1.1 The Cnidarians: *Velella*, *Porpita*, *Physalia*, and *Actinecta*

Numerous floating cnidarians live at the ocean's surface, some famous (or infamous) and others rarely seen. Species like *Velella* sp. (by-the-wind sailor) and *Porpita* sp. (blue button) are central to the surface food web (main text Fig. 3). They possess symbiotic dinoflagellates in their tissue, and like their benthic coral cousins, these symbionts may allow them to survive in oligotrophic waters. *Velella* and *Porpita* are the only two genera of the chondrophore clade within Hydrozoa, and likely evolved convergently with another neustonic Hydrozoan genera: *Physalia* (Portuguese man-o-war). Both *Physalia* and *Velella* poses "sails," which allow them to travel based on wind direction [1]; *Physalia* also utilizes trailing tentacles that serve as a sea anchor on in the open ocean [2], and pack a powerful sting. Sea anemones in the genus *Actinecta* are rarely seen, but also float submerged on the ocean's surface, similar to *Porpita*, but using a bubble float on the pedal disc.

1.1.2 The mollusks *Janthina*, *Recluzia*, and *Glaucus*

Bubble rafting snails *Recluzia* and *Janthina* construct floating rafts by dipping their anterior foot into the water's surface and wrapping trapped air in a layer of mucus to form a bubble, which they then adhere to a raft. The enigmatic *Recluzia* feeds upon the sea anemone *Actinecta*, and both are brown-yellow in color. In contrast, the violet snails

Janthina prey on *Velella*, *Porpita*, and sometimes *Physalia* [3], though they cannot move or hunt. Instead, *Janthina* rely on passive contact with their prey.

Other species include the nudibranch *Glaucus* (blue sea dragon), which also feeds on floating hydrozoans [3] and swallows air to stay afloat. There are multiple cryptic species of *Glaucus* [4], and species in this genus may show a high degree of regional isolation [5].

1.1.3 The Crustacean: *Dosima*

The only truly neustonic barnacle, *Dosima fascicularis* (Buoy barnacle) lives at the ocean's surface by first attaching to floating objects as larvae (such as feathers), and secreting an airy pillow-like float rather than the normal hard cement used by other barnacles [6, 7]. This float allows *Dosima* barnacles to eventually outgrow their larval home and drift independently.

1.1.4 The Algae: *Sargassum*

Neustonic seaweeds like *Sargassum fluitans* and *Sargassum natans* have numerous gas-filled floats to remain at the ocean's surface. These algae create habitat for a variety of *Sargassum*-associated species, particularly at the western edge of the North Atlantic Subtropical Gyre, known as the Sargasso Sea [8]. In the Pacific the algal genus *Turbinaria* reproduces with floating fronts [9].

In addition, over 20 species of algal have been found floating at the surface, and eight species of sea grass [10].

1.1.5 Microorganisms

Diverse microorganisms occupy the ocean's surface ecosystem [11–15], and may play a significant role in gas exchange between the ocean and atmosphere [16]. Many of these organisms have been identified from the sea-surface microlayer, which, depending on definition, extends from 100-1000µm below the ocean's surface [11]. The ocean's surface has unique chemical and physical properties that may concentrate species specifically adapted to these conditions. For example, bacterioneuston living in the sea surface microlayer are often brightly colored [17], possibly as protection against solar radiation. The surface microlayer may be largely dominated by heterotrophic organisms, including both bacteria and microeukaryotes, which take advantage of surface associated compounds [13]. Other species may extend beyond the sea-surface microlayer but still associated with the surface, including the ecologically important cyanobacterium *Trichodesmium* [18]. Still, as with larger organisms, surface microorganisms are generally poorly known (e.g. [15]).

1.2 Organisms that live on water surface tension (Epineuston)

1.2.1 The only true open-ocean insects *Halobates*

Epineustonic organisms live on the water's surface, and in the open ocean all epineustonic species belong to the insect genus *Halobates*. Known as 'sea skaters,' *Halobates* sp. prey on other neustonic species and zooplankton trapped at the surface [19]. *Halobates* lay eggs on a variety of objects, including floating feathers, wood, plastic etc. [20], and, unusually, on pelagic molluscs like *Atlanta turriculata* [21].

1.3 Mobile organisms living below the surface (Hyponesuton)

1.3.1 Copepods

A wide variety of copepods can be found at the ocean's surface [22–29]. Some neustonic copepods possess remarkable adaptations, especially within the Pontellid copepods. Pontellid adaptations include specialized surface attachment structures [30], blue pigmentation [22, 26], and even flying behavior to escape predators [28]. Sapphirinidae copepods are often also associated with the surface [27], and some species have incredible structural coloration [31]. As in many marine ecosystems, copepods represent a major food source for a variety of neustonic and surface-associated species.

1.3.2 Isopods

Idotea metallica is a remarkable surface-associated isopod, that can be found either floating upside down on the ocean's surface (personal observation) or attached to floating debris or neuston (such as the bubble rafts of *Janthina*). It is commonly associated with flotsam [32], and is capable of actively swimming from one floating object to another. This species ostensibly occurs globally in warm waters, though as with many surface-associated species, information on its genetic diversity is scares. It is often flushed into more northern regions by shifting currents. For individuals arriving in the summer months in Helgoland (Germany; North Sea) the fundamental thermal niche is 16°C, with the coldest tolerable temperature likely around 13°C [33]. However, these thermal tolerance limits should be considered with caution: like many neustonic species, *I. metallica* is poorly studied, and whether it is truly one species or many cryptic species is unclear.

1.3.3 Shrimp

Several species of shrimp are associated with floating *Sargassum*, and may also be found swimming at the surface, including *Latreutes fucorum* and *Hippolyte coerulescens*. Neustonic shrimp exhibit a remarkable array of color patterns [34], including the common neustonic blue, with chromophores that can respond to changing light conditions [35].

1.3.4 Fish

A remarkable diversity of fish spend their early life at the ocean's surface (Table 1). This list includes many well-known, ecologically, and economically important species from a variety of habitats. Pelagic fish species include some anchovy, mahi-mahi, marlins, swordfish, amberjack and Atlantic mackerel. Well-known and ecologically important benthic fish associate with the surface when young, including species of: lefteye flounder, blenny, goby, and seahorse/seadragon/pipefish. Deep-sea fish with surface larvae include viperfish and lanternfish. Many eels, both reef, benthic, and deep-sea, nocturnally migrate to the surface layer as larvae [36]. But while the ocean's surface may seem like an odd habitat for larval deep-sea fish, they are far from the most unusual. Diverse fish that migrate into freshwater as adults (either as a habitat or spawning ground) rely on the neuston when young. Yearling and sub-yearling salmon of various species consume neustonic prey in the Northern California Current [37]. American European swim from their freshwater rivers and converging in the middle of the North Atlantic to spawn in the Sargasso Sea. Some fish occupy the ocean's surface for their whole lives, and are even capable of soaring above the waves, including flying fish and halfbeaks. Others frequent the ocean's surface, including basking species like sunfish and basking sharks.

1.3.5 Cephalopod

While no cephalopod is confined to the surface layer permanently, some frequent the surface habitat and are adapted to utilize it. Female argonaut octopus (*Argonauta* spp.) dip their paper-like shell into the air, trapping gas bubbles that they then use to maintain buoyancy [38, 39]. Diverse flying squid species in the Ommastrephidae and Onychoteuthidae can launch themselves from the water and soar for impressive distances, some can reach highs of over 3 meters and others can sail for distances up to 55 meters [40].

1.4 Rafting organisms

While this Essay focuses on free-living organisms at the ocean's surface, the ocean's surface ecosystem also includes rafting species. These species live either attached to neustonic organisms (e.g. barnacles that settle on *Janthina* shells) or inanimate debris. Some rafting species have evolved to live on debris at the ocean's surface, such as the smooth gooseneck barnacle *Lepas anatifera*, while others may be coastal species that settle on near-shore floating debris and are then transported by currents to the open ocean. Several excellent reviews cover the biology of rafters, including the floating substrata of rafters [41], the rafting community [42], and the biogeographical and evolutionary consequences of rafting [43].

References

1. Ferrer L, Pastor A. The Portuguese man-of-war: Gone with the wind. Regional Studies in Marine Science. 2017;14:53–62.

2. Iosilevskii G, Weihs D. Hydrodynamics of sailing of the Portuguese man-of-war Physalia physalis. Journal of The Royal Society Interface. 2009;6(36):613–626.

3. Bieri R. Feeding preferences and rates of the snail, Ianthina prolongata, the barnacle, lepas anserifera, the nudibranchs, glaucus atlanticus and fiona pinnata, and the food web in the marine neuston. Publications of the Seto Marine Biological Laboratory. 1966;14(2):161–170.

4. Churchill CKC, Valdés Á, Ó Foighil D. Molecular and morphological systematics of neustonic nudibranchs (Mollusca : Gastropoda : Glaucidae : Glaucus), with descriptions of three new cryptic species. Invertebrate Systematics. 2014;28(2):174–22.

5. Churchill CKC, Valdés Á, Ó Foighil D. Afro-Eurasia and the Americas present barriers to gene flow for the cosmopolitan neustonic nudibranch Glaucus atlanticus. Marine Biology. 2014;161(4):899–910.

6. Zheden V, Klepal W, von Byern J, Bogner FR, Thiel K, Kowalik T, et al. Biochemical analyses of the cement float of the goose barnacle Dosima fascicularis– a preliminary study. Biofouling. 2014;30(8):949–963.

7. Zheden V, Kovalev A, Gorb SN, Klepal W. Characterization of cement float buoyancy in the stalked barnacle Dosima fascicularis(Crustacea, Cirripedia). Interface Focus. 2015;5(1):20140060–7.

8. Coston-Clements L, Settle LR, Hoss DE, Cross FA. *Utilization of the Sargassum habitat by marine invertebrates and vertebrates, a review*. vol. 296. US Department of Commerce, National Oceanic and Atmospheric Administration,

National Marine Fisheries Service, Southeast Fisheries Science Center, Beaufort Laboratory.; 1991.

9. Stewart HL. Ontogenetic changes in buoyancy, breaking strength, extensibility, and reproductive investment in a drifting macroalga turbinaria ornata (Phaeophyta). Journal of Phycology. 2006;42(1):43–50.
10. Thiel M, Gutow L. The ecology of rafting in the marine environment. I. The floating substrata. Oceanography and Marine Biology - An Annual Review. 2005;42:181–264.
11. Marshall HG, Burchardt L. Neuston: Its definition with a historical review regarding its concept and community structure. Archiv für Hydrobiologie. 2005;164(4):429–448.
12. Franklin MP, McDonald IR, Bourne DG, Owens NJP, Upstill-Goddard RC, Murrell JC. Bacterial diversity in the bacterioneuston (sea surface microlayer): the bacterioneuston through the looking glass. Environmental Microbiology. 2005;7(5):723–736.
13. Sieburth JM, Willis PJ, Johnson KM, Burney CM, Lavoie DM, Hinga KR, et al. Dissolved organic matter and heterotrophic microneuston in the surface microlayers of the north atlantic. Science. 1976;194(4272):1415–1418.
14. Taylor JD, Cunliffe M. High-throughput sequencing reveals neustonic and planktonic microbial eukaryote diversity in coastal waters. Journal of Phycology. 2014;50(5):960–965.
15. Rahlff J. A review on viral–bacterial associations at air–water interfaces. Viruses. 2019;11(2):191–13.
16. Upstill-Goddard RC, Frost T, Henry GR, Franklin M, Murrell JC, Owens NJP. Bacterioneuston control of air-water methane exchange determined with a laboratory gas exchange tank. Global Biogeochemical Cycles. 2003;17(4):1–15.
17. Tsyban AV. Marine bacterioneuston. Journal of Oceanographical Society of Japan. 1971;27(2):56–66.
18. Capone DG, Zehr JP, Paerl HW, Bergman B, Carpenter EJ. Trichodesmium, a Globally Significant Marine Cyanobacterium. Science. 1997;276(5316):1221–1229.
19. M ller Andersen N, Cheng L. The Marine Insecthalobates(Heteroptera: Gerridae). In: Oceanography and Marine Biology. CRC Press; 2010. p. 119–179.
20. Cheng L. Biology of Halobates (heteroptera: gerridae) . Annual Review of Entomology. 1985; p. 111–135.
21. Andersen NM, Cheng L. The marine insect Halobates (Heteroptera: Gerridae): biology, adaptations, distribution, and phylogeny. Oceanography and Marine Biology - An Annual Review. 2004;42:119–180.
22. Herring PJ. Blue pigment of a surface-living oceanic copepod. Nature. 1965;205(4966):103–104.
23. Smith J, Richter C, Fabricius K, Cornils A. Neustonic copepods (Labidocera spp.) discovered living residentially in coral reefs. Marine Biodiversity. 2017;49(1):345–355.

24. Ianora A, Santella L. Diapause embryos in the neustonic copepodAnomalocera patersoni. Marine Biology. 1991;108:389–349.

25. Jeong HG, Suh HL, Yoon YH, Choi IH, Soh HY. The first records of two neustonic calanoid copepods, pontella securifer and P. sinica (Calanoida, Pontellidae) in the south sea, Korea. Ocean Science Journal. 2008;43(2):91–100.

26. Rahlff J, Ribas-Ribas M, Brown SM, Mustaffa NIH, Renz J, Peck MA, et al. Blue pigmentation of neustonic copepods benefits exploitation of a prey-rich niche at the air-sea boundary. Scientific Reports. 2018; p. 1–6.

27. Zaitsev Y. Neuston of seas and oceans. In: The Sea Surface and Global Change. Cambridge University Press; 2009. p. 371–382.

28. Svetlichny L, Larsen PS, Kiørboe T. Swim and fly: escape strategy in neustonic and planktonic copepods. Journal of Experimental Biology. 2018;221(2):jeb167262–9.

29. Nair SR, Achuthankutty CT, Haridas P, Nair V. Neuston composition in central Arabian Sea. Indian Journal of Marine Sciences. 1989;18:155–159.

30. Ianora A, Miralto A, Vanucci S. The surface attachment structure: a unique type of integumental formation in neustonic copepods. Marine Biology. 1992;113(3):401–407.

31. Chae J, Nishida S. Integumental ultrastructure and color patterns in the iridescent copepods of the family Sapphirinidae (Copepoda: Poecilostomatoida). Marine Biology. 1994;119:205–210.

32. Abelló P, Guerao G. Distribution of the neustonic isopod Idotea metallica in relation to shelf-slope frontal structures. Journal Of Crustacean Biology. 2004;24(4):558–566.

33. Gutow L, Franke HD. On the current and possible future status of the neustonic isopod Idotea metallica Bosc in the North Sea: a laboratory study. Journal of Sea Research. 2001;45(1):37–44.

34. Hacker SD, Madin LP, 1991. Why habitat architecture and color are important to shrimps living in pelagic Sargassum: use of camouflage and plant-part mimicry. Marine ecology progress series. 1991; p. 143–155.

35. Brown F. The coloration and color changes of the gulf-weed shrimp, Latreutes fucorum. The American Naturalist. 1939;73(The Coloration and Color Changes of the Gulf-Weed Shrimp,):564–568.

36. Miller M. Ecology of anguilliform leptocephali: remarkable transparent fish larvae of the ocean surface layer. Aqua-BioScience Monographs. 2009;2(4):1–94.

37. Brodeur RD, Pool SS, Fish TMNPA, 2013. Prey selectivity of juvenile salmon on neustonic mesozooplankton in the northern California Current. Journal of Ichthyology. 2013;.

38. Finn JK, Norman MD. The argonaut shell: gas-mediated buoyancy control in a pelagic octopus. Proceedings of the Royal Society B: Biological Sciences. 2010;277(1696):2967–2971.

39. Dall WH. Notes on the Argonaut. The American Naturalist. 1869;3(5):236–239.

40. Maciá S, Robinson MP, molluscan PCJo, 2004. New observations on airborne jet propulsion (flight) in squid, with a review of previous reports. Journal Of Crustacean Biology. 2004;.

41. Thiel M, Gutow L. The ecology of rafting in the marine environment. I. the floating substrata. In: Gibson RN, Atkinson RJA, Gordon JDM, editors. Oceanography and Marine Biology: An Annual Review. CRC Press; 2004. p. 181–264.

42. Thiel M, Gutow L. The ecology of rafting in the marine environment. II. The rafting organisms and community. vol. 43. Centro de Estudios Avanzados en Zonas Áridas (CEAZA), La Serena, Chile; 2005.

43. Thiel M, Haye PA. The ecology of rafting in the marine environment. III. Biogeographical and evolutionary consequences. Oceanography and Marine Biology - An Annual Review. 2006;44:323–429.
